# Polyadenylation and nuclear export of mRNAs

**DOI:** 10.1074/jbc.REV118.005594

**Published:** 2019-01-25

**Authors:** Murray Stewart

**Affiliations:** From the MRC Laboratory of Molecular Biology, Francis Crick Avenue, Cambridge Biomedical Campus, Cambridge CB2 0QH, United Kingdom

**Keywords:** nuclear transport, polyadenylation, RNA splicing, nuclear pore, RNA helicase, RNA binding protein, gene expression pathway, nuclear posttranscriptional modification, ribonuclear protein (RNP), spliceosome

## Abstract

In eukaryotes, the separation of translation from transcription by the nuclear envelope enables mRNA modifications such as capping, splicing, and polyadenylation. These modifications are mediated by a spectrum of ribonuclear proteins that associate with preRNA transcripts, coordinating the different steps and coupling them to nuclear export, ensuring that only mature transcripts reach the cytoplasmic translation machinery. Although the components of this machinery have been identified and considerable functional insight has been achieved, a number of questions remain outstanding about mRNA nuclear export and how it is integrated into the nuclear phase of the gene expression pathway. Nuclear export factors mediate mRNA transit through nuclear pores to the cytoplasm, after which these factors are removed from the mRNA, preventing transcripts from returning to the nucleus. However, as outlined in this review, several aspects of the mechanism by which transport factor binding and release are mediated remain unclear, as are the roles of accessory nuclear components in these processes. Moreover, the mechanisms by which completion of mRNA splicing and polyadenylation are recognized, together with how they are coordinated with nuclear export, also remain only partially characterized. One attractive hypothesis is that dissociating poly(A) polymerase from the cleavage and polyadenylation machinery could signal completion of mRNA maturation and thereby provide a mechanism for initiating nuclear export. The impressive array of genetic, molecular, cellular, and structural data that has been generated about these systems now provides many of the tools needed to define the precise mechanisms involved in these processes and how they are integrated.

## Introduction

In eukaryotes, the nuclear envelope functions to separate transcription from translation, and this enables transcripts to be modified substantially in the nucleus before they are exported to the cytoplasm where they are translated. During these nuclear steps in the gene expression pathway, pre-RNA transcripts are modified by the addition of 5′ caps and 3′ poly(A) tails, and frequently introns are removed to generate mature mRNAs. A broad spectrum of ribonuclear proteins are bound to transcripts during the nuclear processing (for reviews, see Refs. 1 and 2) that ultimately generates mature export-competent messenger ribonuclear particles (mRNPs)^2^ that are exported to the cytoplasm through the nuclear pores that perforate the nuclear envelope and mediate the movement of macromolecules between the cytoplasmic and nuclear compartments. The nuclear processing steps in the gene expression pathway are crucial to ensuring that the coding sequence of the gene is delivered to ribosomes to generate the appropriate proteins, as well as regulating mRNA stability and mediating translation. Errors or defects in the nuclear transcript modification steps or in mRNA nuclear export frequently result in impaired grow or death of cells and are associated with a broad range of different disease conditions. Consequently, these processes are closely coordinated and are coupled to the nuclear export of mRNA to ensure that only mature and completely processed transcripts reach cytoplasmic ribosomes for translation into proteins.

The transport of macromolecules, such as proteins and RNAs, between the cytoplasmic and nuclear compartments is mediated by nuclear pores that are huge macromolecular assemblies that span the nuclear envelope. Nuclear transport is an active, energy-requiring process and is highly selective. Because the pores function as a barrier to the passive diffusion of molecules larger than ∼40 kDa, only macromolecules that are bound to specific carrier molecules (“transport factors”) are able to move through them.

### Nuclear pore structure and function

Considerable progress has been made on establishing the structure of nuclear pores and relating this to their function. Nuclear pores are constructed from multiple copies of upward of 30 different proteins that are arranged to generate a cylindrical structure that has 8-fold rotational symmetry and that has a central transport channel through which macromolecules move (for reviews, see Refs. 3–5). The individual proteins from which nuclear pores are constructed are collectively referred to nucleoporins, or “nups” for short, and are frequently distinguish by a number that reflects their *M*_r_ (such as, for example, Nup42 or Nup153). Although there is considerable sequence variation between species, the overall architecture of nuclear pores is generally conserved; albeit budding yeast nuclear pores are somewhat smaller than their metazoan counterparts (3–5). Crystal structures of many individual components and the subcomplexes they form have been obtained and, together with recent cryo-EM structures of intact pores, have provided a detailed model of how they are arranged to generate the cylindrical skeleton of the pore (for reviews, see Refs. 3–5). There are also fibrous assemblies protruding from both faces of the pore. In the nucleus, these fibers form a nuclear basket, whereas at the cytoplasmic face they form a series of filaments that extend into the cytoplasm.

The architecture of nuclear pores has provided a basis for understanding the mechanism by which nuclear pores generate a barrier to the passive diffusion of large macromolecules while facilitating the selective active transport of specific cargoes. In addition to the structured protein domains that constitute the nuclear pore skeleton, many nucleoporins also contain natively unfolded regions that lack defined secondary structure. These regions fill the central channel through which macromolecules are transported and generate a barrier that impairs movement through the pores of macromolecules larger than ∼40 kDa. These natively unfolded regions generally contain tandem sequence repeats that contain hydrophobic cores rich in phenylalanine and glycine, with sequences such as GLFG and F*X*FG, separated by linkers that are generally hydrophilic, and are referred to as FG-nucleoporins (“FG-nups”). The transport barrier generated by these densely packed regions of FG-nups in the transport channel can be overcome by a range of nuclear transport factors (“carriers”) that mediate the import and export of specific macromolecular cargoes (6, 7). There is a range of different models for how the FG-nups generate barrier function, including entropic effects due to molecular crowding related to the formation of molecular brushes (8–10) and cohesion between nucleoporins (11). These mechanisms are not mutually exclusive, and all may contribute to impeding the movement of macromolecules through the pore channel. Transport factors are able to overcome the barrier function by transiently interacting with the hydrophobic cores of the FG-nups and so enabling cargo:carrier complexes to passively diffuse rapidly back and forth through the pores. Although this interaction enables movement back and forth through the pores, energy is required to impose directionality and generate vectorial transport. Therefore, nuclear transport is different from simple passive diffusion through a pore in which movement is dictated simply by the chemical potential. Instead, metabolic energy is used to rectify simple diffusion so that the direction of transport does not depend on the difference in chemical potential of the cargo in the donor and acceptor compartments. In this way, nuclear transport facilitates the generation of the distinctive compositions of the nuclear and cytoplasmic compartments.

### Overview of nuclear transport

**Figure 1.**
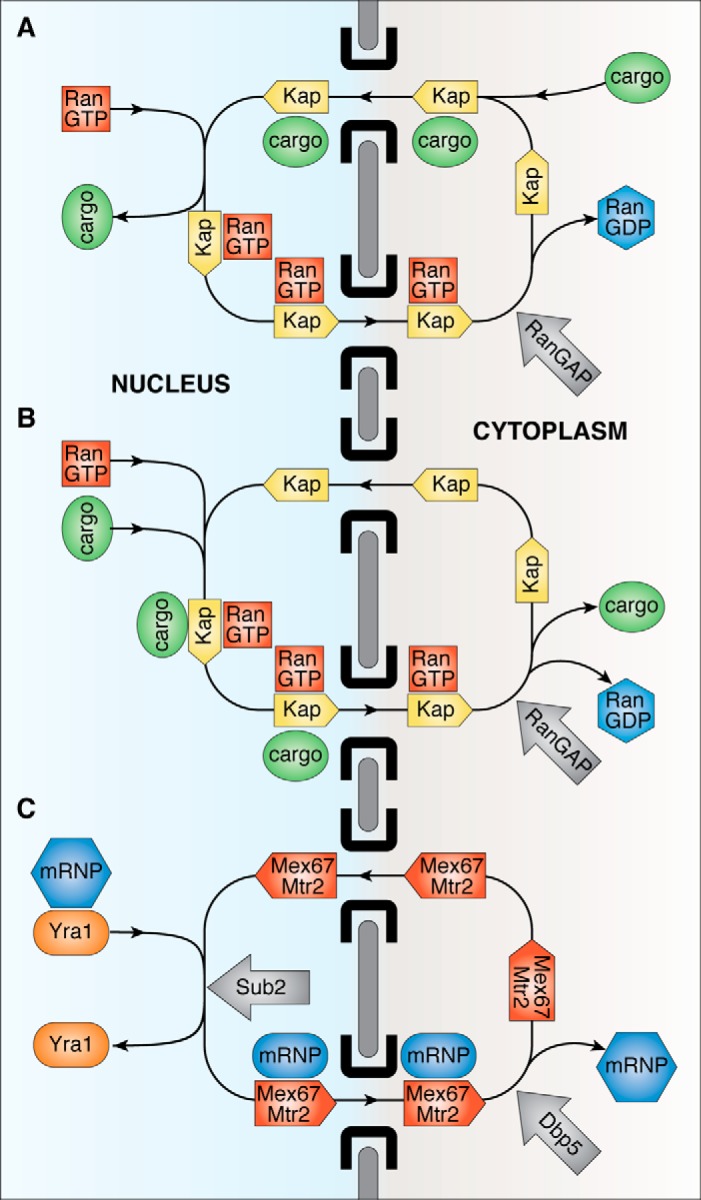
**Overview of nuclear transport pathways for macromolecules.** These pathways are all based on a thermal ratchet mechanism in which energy is used to rectify Brownian motion by mediating the assembly of cargo:carrier complexes in the donor compartment and their disassembly in the acceptor compartment. Proteins and small RNAs are transported using karyopherin-based carriers to mediate movement through nuclear pores. In nuclear protein import (*A*) karyopherins (*Kap*; *yellow*) such as importin-β bind their cargoes (*green*) in the cytoplasm (often employing an adapter such as importin-α), and then, when the cargo:carrier complex reaches the nucleus, RanGTP (*red*) binding to the karyopherin dissociates the cargo, after which the karyopherin:RanGTP complex returns to the cytoplasm where the RanGTPase is activated by RanGAP, generating RanGDP that dissociates from the karyopherin, freeing it for a further import cycle. The RanGDP is then recycled to the nucleus where RanGEF (RCC1, Prp20) recharges it with GTP. The export of proteins and small RNAs (*B*) is mediated by an analogous pathway, except that here the cargo binds to the karyopherin (such as Crm1) complexed with RanGTP in the nucleus and is released in the cytoplasm following GTP hydrolysis. The export of mRNAs (*C*) employs a different pathway that uses the Mex67:Mtr2 (NXF1:NXT1) complex (Fig. 3) as a carrier to which binding in the nucleus and release in the cytoplasm are mediated by DEAD-box helicases (Sub2 and Dbp5, respectively) that hydrolyze ATP to remodel the mRNP. An additional feature of mRNA export is that it is necessary for the nuclear steps of the gene expression pathway to have been completed so that only fully matured transcripts are exported for translation in the cytoplasm. The machinery involved in these steps is summarized in Fig. 2.

Although nuclear transport is an active energy-requiring process, the movement of cargo:carrier complexes through the nuclear pore transport channel itself is mediated by simple passive diffusion-based Brownian motion. The energy that enables transport independent of the chemical potential of the cargoes in each compartment is provided indirectly through the assembly and disassembly of the cargo:carrier complexes, which for the transport of protein cargoes is mediated by the Ras-family GTPase, Ran (Gsp1 in budding yeast). For example, in nuclear protein import (for reviews, see Refs. 6 and 7), karyopherin-β–family carriers such as importin-β (Kap95 in yeast) bind cargo proteins in the cytoplasm, and after passage through the pores to the nucleus, the cargo:carrier complex is dissociated by RanGTP binding to the karyopherin carrier, releasing the cargo (Fig. 1*A*). The carrier:RanGTP complex then returns to the cytoplasm where the Ran GTPase-activating protein (RanGAP) catalyzes GTP hydrolysis, generating RanGDP, which dissociates from the karyopherin, freeing it for a further import cycle. The RanGDP then returns to the nucleus (using NTF2 as a carrier (12)) where the Ran guanine nucleotide exchange factor (RanGEF), RCC1 (Prp20 in yeast), recharges it with GTP. Analogous machinery is used for the nuclear export of proteins and small RNAs, although here cargo:carrier assembly in the nucleus requires β-karyopherins, such as CRM1 (Xpo1), to be bound to RanGTP, with the cargo being released following GTP hydrolysis in the cytoplasm (Fig. 1*B*). The net result of these cycles is that the energy derived from GTP hydrolysis is used to impose directionality on transport by rectifying the thermal diffusion (Brownian motion) of the components of the transport machinery by facilitating assembly of the cargo:carrier complex in the donor compartment and its disassembly in the target compartment and so is an example of a Brownian ratchet mechanism (13). This mechanism enables transport to be independent of the concentration or chemical potential of cargoes in each compartment and so facilitates the different macromolecular compositions of the nucleus and cytoplasm. Although they do not contribute directly to the energetics of the transport cycle and the relative concentrations of cargoes in each compartment, several nucleoporins do contribute to the kinetics of transport by concentrating material at one face of the nuclear pores. For example, Nup1 binding helps concentrate Kap95 complexes and RanGTP at the nuclear face of the pore (14), and Nup358 appears to perform an analogous function at the cytoplasmic face through binding RanGAP (15).

### Nuclear export of mRNA

**Figure 2.**
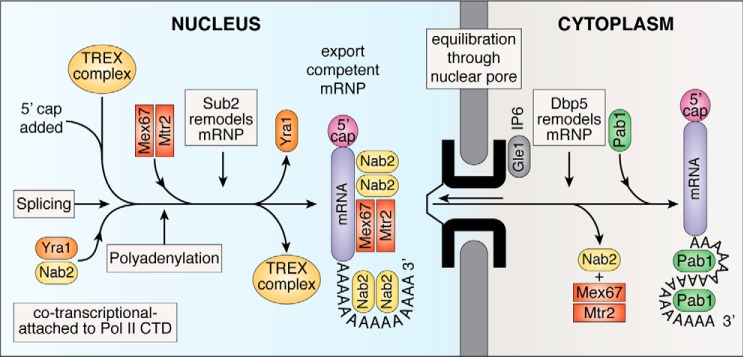
**Outline of the gene expression pathway from transcription to translation for budding yeast.** Transcripts are modified by the addition of 5′ caps and 3′ poly(A) tails and the splicing out of introns if present before a structural rearrangement, mediated by Sub and the TREX complex, removes Yra1 and attaches Mex67:Mtr2 to generate an export-competent mRNP to which Nab2 is also bound. The precise nature of the structural rearrangement remains to be established but may involve the generation of RNA hairpins that bind more strongly to the transport factor. The mRNP can then diffuse back and forth through nuclear pores as a result of interactions between Mex67:Mtr2 and FG-nucleoporins overcoming the barrier function of the pore. At the cytoplasmic face of the pore, the DEAD-box helicase Dbp5, working in conjunction with Nup42 and Gle1, remodels the RNA to release Mex67:Mtr2 and Nab2, thereby preventing the mRNA from returning to the nucleus. Pab1 also replaces Nab2 on the poly(A) tail. Although this sequence of processing steps tends to resemble a production line, the steps may not necessarily occur in a defined sequence, and nuclear export, the culmination of the nuclear phase of the gene expression pathway, appears to only require that all steps have been completed successfully. Metazoans have an analogous pathway but differ in some details, primarily in the addition of the EJC immediately after the 3′ splice site when exons are joined. In metazoans, the first EJC together with the 5′ cap facilitates the binding of the TREX complex and subsequent attachment of the Mex67:Mtr2 homologue NXF1:NXT1. *IP6*, inositol hexaphosphate; *Pol II CTD*, polymerase II C-terminal domain.

Although some small mRNAs and virus RNAs are exported using CRM1 (through binding of adapter proteins such as HIV REV), the overall mechanism of nuclear export of most mRNA is conserved within eukaryotes and employs an analogous thermal ratchet mechanism (Fig. 1*C*), although here the carriers are not members of the karyopherin-β family, and the energy used to rectify movement through the assembly and disassembly of the cargo:carrier complexes is provided by ATP hydrolysis on DEAD-box helicases (16–20). Transcripts are only exported when a complex series of steps in the nuclear segment of the gene expression pathway have been completed (Fig. 2), which entails a considerable level of coordination and relies on a complex signaling network to impair improperly or incompletely processed mRNAs reaching the translation machinery in the cytoplasm.

**Figure 3.**
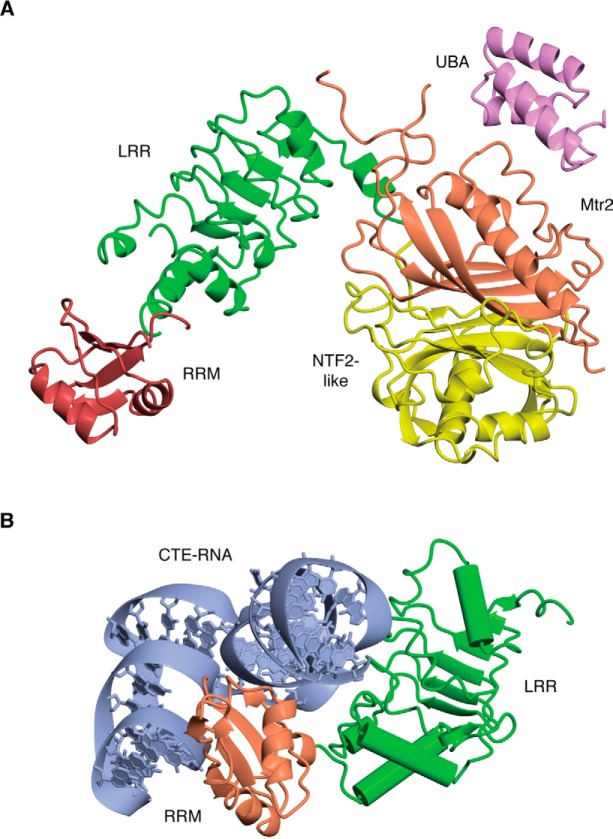
**Mex67:Mtr2/NXF1:NXT1 structure and interaction with CTE-RNA.** *A*, domain structure of the *S. cerevisiae* Mex67:Mtr2 complex. The metazoan homologue, NXF1:NXT1 (also called TAP:P15), has a similar structure but has an additional unstructured arginine-rich domain at its N terminus. Mex67 contains four structural modules (RRM domain, LRR domain, NTF2-like domain, and UBA domain) that are connected by flexible linkers. Mtr2 also has an NTF2-like fold and binds to the Mex67 NTF2-like domain to form a heterodimer. The RRM, LRR, and NTF2-like domains bind to RNA, whereas the NTF2-like and UBA domains interact with FG-nucleoporins (based on Protein Data Bank (PDB) codes 1OAI and 4WWU). *B*, complex formed between viral CTE-RNA and the RRM and LRR domains of TAP (the human homologue of Mex67) showing the secondary structure of the RNA (based on PDB code 3RW6) that forms a bent hairpin (*blue*). Similar RNA secondary structures may be generated in the nucleus by helicases to facilitate the binding of Mex67:Mtr2 to generate export-competent mRNPs.

The budding yeast Mex67:Mtr2 complex (Fig. 3*A*) and the homologous metazoan NXF1:NXT1 (also called TAP:P15) complex function as general mRNP export factors to mediate the movement of mature mRNPs through nuclear pores to the cytoplasm (21, 22). However, Mex67:Mtr2 and NXF1:NXT1 bind RNA nonspecifically, and so additional factors are needed to mediate their attachment to mRNAs. The binding of Mex67:Mtr2 to mature mRNA in the nucleus is mediated by DEAD-box helicases such as Sub2 (UAP56 in metazoans) and possibly Dbp2 (23, 24), whereas disassembly of the complex when it reaches the cytoplasmic face of the pore is mediated by the DEAD-box helicase Dbp5 (vertebrate DDX19). Both the attachment and detachment of Mex67:Mtr2 to mRNAs are probably mediated through generating conformational changes in mRNA structure (17, 18, 20). Both of these remodeling processes also involve a number of accessory proteins, including the TREX complex and Yra1/ALYREF in the nucleus and Gle1 and Nup42 in the cytoplasm (25–38). After the transport factor has been dissociated from the mRNA, it is imported back through nuclear pores to participate in another mRNA export cycle. The actual transport of mRNPs through nuclear pores is rapid compared with the time taken at the nucleoplasmic and cytoplasmic faces to assemble and disassemble export-competent mRNPs and associated quality control processes (39, 40).

Yeast Mex67 is constructed from four domains: an N-terminal RRM domain followed by a LRR domain, an NTF2-like domain that binds Mtr2 (which also has an NTF2 fold), and finally a C-terminal UBA-fold domain (Fig. 3). NXF1 has a similar architecture with an additional N-terminal arginine-rich RNA-binding domain (RBD) (41). The UBA domain and the NTF2 domain heterodimer bind to FG-nups, whereas the RBD, RNP, and LRR domains bind to RNA (20). The DEAD-box helicase Dbp5 removes Mex67:Mtr2 from transcripts at the cytoplasmic face of the pores and probably also removes other factors such as Nab2 (a factor that helps coordinate the nuclear steps as well as regulating poly(A) tail length) from the mRNPs (29, 31, 35, 42). The activity of Dbp5 is enhanced greatly by nuclear pore components Gle1 and Nup42 and by inositol hexaphosphate as well as Gfd1, a protein that also binds to Nab2 and that is thought to accompany it to the cytoplasm (25, 36). DBP5 and GLE1 function to remove NXF1:NXT1 from metazoan transcripts in an analogous manner.

Yra1 and Sub2, working in conjunction with the TREX complex, are necessary to generate mature, export-competent transcripts in the nucleus of budding yeast (20, 28, 32, 38). Yra1 acts as an adapter, facilitating Mex67 attachment to the transcript, but paradoxically is removed before export (29), probably as a result of the TREX complex (especially the DEAD-box helicase Sub2/UAP56) orchestrating a remodeling of the transcript that likely involves a change in mRNA secondary structure (18, 19, 43), together with Yra1 ubiquitinylation by the Tom1 E3 ligase (44). ALYREF functions as an analogous adapter for TAP:P15 in metazoans. In addition, RNA-binding proteins rich in Ser and Arg (SR proteins), such as Npl3, may also function as adapters for the Mex67:Mtr2 complex in some instances (45).

The TREX complex, which is conserved between yeast and metazoans, contributes to the integration of the nuclear steps of the gene expression pathway and nuclear export (32). The yeast TREX complex is primarily associated with the transcription machinery and is based on a THO complex core, consisting of Tho2, Hpr1, Mft1, and Thp2, to which Yra1 and Sub2 become attached. In metazoans, however, TREX appears instead to associate primarily with the splicing machinery through its binding to the exon-junction complex (EJC) that is deposited ∼20 nucleotides upstream of the most 5′ exon–exon junction (46). At a later stage, the TREX complex contributes to the generation of export-competent mRNPs; albeit this process appears to be more complex in metazoans and may also involve relieving an autoinhibition based on the arginine-rich N-terminal of NXF1 together with additional components of the TREX complex (41, 43).

A hypothesis for the function of these DEAD-box helicases in rectifying the Brownian motion of the Mex67:Mtr2:mRNA complex has envisaged two reciprocal remodeling steps (7, 19), perhaps in some ways analogous to the remodeling mediated by DEAD/H-box helicases during splicing (47, 48). In the first step of such a mechanism, Sub2/UAP56 hydrolyzes ATP to generate the mRNA conformation to which the NXF1:NXT1/Mex67:Mtr2 export factor binds in the nucleus, thereby generating an export-competent mRNP that can then diffuse back and forth through the nuclear pore transport channel. Once in the cytoplasm, the complex encounters Dbp5 that, in conjunction with Gle1 and Nup42, hydrolyzes ATP to again remodel the complex and revert to the initial RNA conformation, facilitating release of the export factor and thereby preventing return of the transcript to the nucleus. NXF1:NXT1/Mex67:Mtr2 is then recycled to the nucleus to participate in a further export cycle.

Little information is available about the precise nature of the remodeling of the RNA that mediates binding of the transport factor in the nucleus and its release in the cytoplasm, although the viral constitutive transport element (CTE) may give some clues to the RNA secondary structure elements that have greater affinity for NXF1:NXT1. The CTE that is found in some unspliced RNAs from simple retroviruses enables them to bypass the normal pathway for generating mature mRNPs and use the NXF1:NXT1 pathway more efficiently than host transcripts for nuclear export. The ∼130-nt CTE RNA has a 2-fold symmetric motif that enables it to bind to NXF1:NXT1 without a requirement for adapter proteins such as ALYREF (Fig. 2*B*). Each motif in the CTE RNA is predicted to form a distinctive L-shaped stem loop, and structural studies have indicated how this motif binds primarily to the RRM, LRR, and NTF2-like domains as well as suggested how NXF1:NXT1 could dimerize to form a platform with its binding sites arranged to bind the two motifs simultaneously and so facilitate rapid nuclear export of the viral RNA (49, 50). It is likely that a similar RNA secondary structure is generated in host transcripts by nuclear DEAD-box helicases to facilitate NXF1:NXT1 binding, and this is probably a metastable conformation that is easily reversed by the DBP5 helicase to release the transport factor in the cytoplasm.

In addition to facilitating processing and coordinating steps in the nuclear processing of transcripts, some of the proteins that become attached to transcripts in the nucleus result in compaction of the mRNP. As a result of this compaction, in electron micrographs of mature yeast mRNPs, the particles are considerably shorter than would be expected if their RNA had an extended conformation (51), which is also consistent with their observed diffusion properties *in viv*o (40). One well-characterized protein associated with compaction is Nab2 in budding yeast (51), which, in addition to binding to and regulating the length of poly(A) tails, also binds to A-rich regions in the coding region (51–53) and, through its potential to form dimers that are bound to different regions of the transcript, could facilitate compaction (54). SR proteins also appear to contribute to compaction of mRNPs (2).

Mature transcripts become concentrated at the nuclear face of the pores before they are exported, and indeed the time mRNPs reside at the nuclear face is considerably longer than that taken to pass through the nuclear pores to the cytoplasm (39, 55). It is thought that proteins located in the nuclear basket, such as Mlp1 in budding yeast and possibly TPR in metazoans, contribute to this process (56). The N-terminal domain of the poly(A)-binding protein Nab2, for example, binds to Mlp1 and so could facilitate the localization of mature transcripts to the nuclear face of the pores (56). In metazoans, the TREX-2 complex, which is based on a core of GANP to which THP1, DSS1, and ENY2 are bound (57), has been proposed to contribute to chaperoning mRNPs generated in processing centers deep in the nucleus to the nuclear pores to facilitate their transport (58, 59).

### Checkpoints

**Figure 4.**
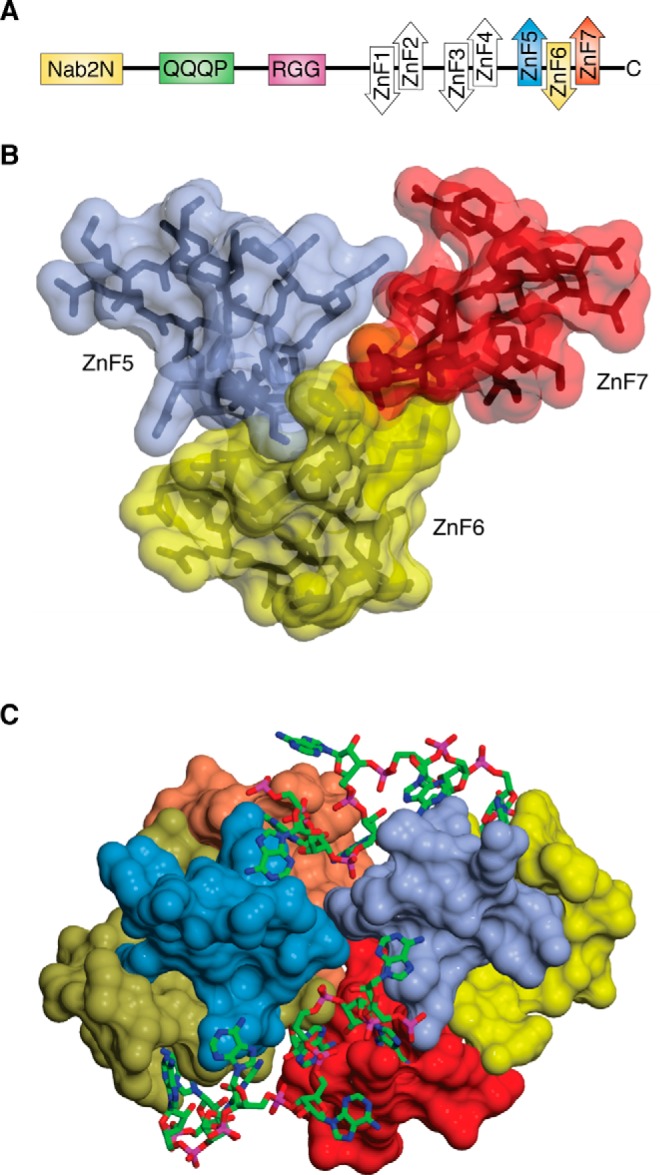
**Nab2 structure and its binding to poly(A) RNA.** *A*, schematic illustration of the domains present in Nab2. The N-terminal Nab2N domain is essential and interacts with Mlp1 and Gfd1. The RGG domain also contains the nuclear localization sequence that is recognized by transportin to return Nab2 to the nucleus after an export cycle. There are seven Zn fingers arranged in three groups (ZnF1+2, ZnF3+4, and ZnF5–7). The fingers within each group interact so that they have a defined orientation to one another. *B*, arrangement of Nab2 Zn fingers 5–7 that impairs binding of a single poly(A) RNA chain to all three simultaneously (based on PDB code 5L2L). *C*, dimerization of Nab2 Zn fingers 5–7 brought about by binding A_11_G RNA (54). The RNA binds to both Nab2 chains to generate the dimer, and it is likely that similar dimers can be formed with full-length Nab2 *in vivo* (based on PDB code 5L2L).

The export of mature transcripts to the cytoplasm for translation represents the culmination of the nuclear phase of the gene expression pathway (Fig. 2). It is important that export-competent mature mRNPs are only generated when splicing and polyadenylation have been completed, and so there are checkpoints that monitor completion of each process. Almost all genes in higher eukaryotes contain introns, and although introns are less common in budding yeast, many highly expressed genes contain introns so that roughly half of the transcripts generated require splicing (for a review, see Ref. 47). Polyadenylation is critical for the stability of most transcripts and so needs to be completed successfully. Although the checkpoints for both polyadenylation and splicing are similar between yeast and higher eukaryotes, there are some differences to accommodate differences in detailed machinery involved, and generally each process tends to be understood in greater detail in yeast. For example, in yeast the Zn-finger poly(A) RNA–binding protein Nab2 (Fig. 4) appears to play a central role in coordinating the nuclear processing and export machinery, but the role of its metazoan analogue ZC3H14 is less well defined (60, 61).

### Splicing

It is crucial that the intervening intron sequences that are present between the exon coding sequences of transcripts are removed by splicing before translation takes place. Most metazoan genes and many yeast genes contain introns, and these are removed in the nucleus by the spliceosome through a complex series of reactions that function to join the 3′ end of one exon to the 5′ end of the next exon (for a review, see Ref. 47). Although the overall splicing mechanism appears to be retained between different eukaryotes (for a review, see Ref. 47), there are some differences in the way in which completion is indicated and how splicing is integrated with mRNP maturation and nuclear export. In metazoans, when each splicing reaction is completed, an exon junction complex (EJC) is deposited close to where the two exons are joined, ∼20–24 nucleotides upstream from the 5′ end of the splice junction, and serves as a platform for interacting with a broad range of other components of the gene expression pathway, both in the nucleus and the cytoplasm. The most 5′ EJC has been proposed to function to recruit the TREX complex that, together with the 5′ cap component CBP80, facilitates binding of NXF1:NXT1 and nuclear export of the mature mRNPs (46). In addition, a broad spectrum of interactions have been demonstrated between the splicing and polyadenylation machineries in both yeast and vertebrate cells (62).

Impeding the nuclear processing and subsequent nuclear export of incompletely or incorrectly spliced transcripts has been proposed to facilitate their elimination by the exosome and has led to a kinetic model of surveillance (62). In yeast, one way in which the export of intron-containing transcripts is impeded involves the splicing and retention complex (63), together with contributions made by Mlp1 and by Nab2, a poly(A)-binding protein that is important in controlling the length of poly(A) tails in *Saccharomyces cerevisiae* (62) that also shows genetic interactions with splicing factors, most notably with the Mud2 component of the U1 small nuclear RNP (61). However, because they fail to copurify after RNase treatment, it is not clear whether Mud2 and Nab2 interact directly or instead may both bind to the same transcript (61). Nab2 is not required for efficient splicing *in vitro*, but *nab2* mutants do show a mild increase in splicing defects *in vivo*, although these do not appear to involve Nab2 having a direct role in splicing itself but rather a quality control function to ensure that only completely spliced transcripts undergo further processing and export (62). Reciprocally, Mud2 deletion generates longer poly(A) tails, reinforcing the evidence for a functional interaction between the splicing and polyadenylation machineries. Moreover, the mammalian analogue of Nab2, ZC3H14, also interacts functionally with a number of spliceosome components (61). Mlp1 and Mlp2, components of the nuclear basket, also function to inhibit the nuclear export of unspliced transcripts, either by retaining them in the nucleus or, alternatively, by accelerating the export of spliced transcripts (64).

### Polyadenylation

Although the lengths of poly(A) tails (∼250 nt in higher eukaryotes but only ∼60 nt in *S. cerevisiae*) and the detailed mechanisms by which length is controlled vary, similar large multisubunit complexes mediate cleavage in the 3′-UTR and subsequent generation of a poly(A) tail in eukaryote transcripts before they are exported to the cytoplasm (65–67). In *S. cerevisiae*, these processes are mediated by the cleavage and polyadenylation factor (CTF) that is organized into three structural modules that mediate key functions: a nuclease module that cleaves the transcript; a polymerase module in which Cft1 binds four other components, including poly(A) polymerase (Pap1) and Fip1; and a phosphatase/APT module that regulates 3′ end processing (65). Metazoans employ the analogous CPSF complex (66, 67). The polyadenylation signal, the sequence motif recognized by the RNA cleavage complex, varies among groups of eukaryotes. Most human polyadenylation sites contain the AAUAAA sequence (68), but this sequence is less common in plants and fungi.

**Figure 5.**
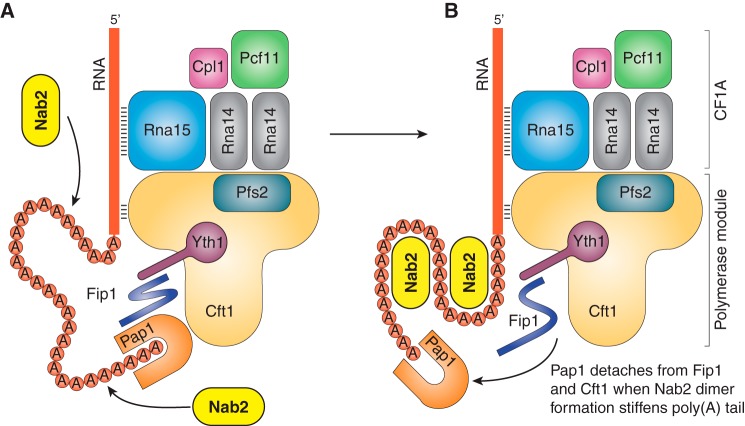
**Model for the regulation of poly(A) tail length by Nab2 in yeast.** Poly(A) tail length in *S. cerevisiae* appears to be regulated by a mechanism analogous to that proposed for the way in which PABPN1 functions in metazoans (66, 67) to terminate polyadenylation by dissociating poly(A) polymerase from the cleavage and polyadenylation machinery (CPSF) so that it ceases to be processive. Each PABPN1 binds to ∼12–15 adenines, and when a sufficient number have been added, they form an approximately spherical aggregate that is proposed to stiffen the poly(A) tail, forcing poly(A) polymerase to dissociate from the CPSF, after which it ceases to be processive and dissociates from the poly(A) tail, terminating polyadenylation. An analogous model for polyadenylation termination in *S. cerevisiae* envisages that following cleavage, poly(A) polymerase (Pap1) synthesizes the poly(A) tail processively but only while it remains attached to the polyadenylation factor (CPF) through binding to Fip1 and Cft1 (for simplicity, the cleavage module has been omitted from the figure). Pap1 holds the growing poly(A) tail at one end while Rna15 and Cft1 hold its other end so that it forms a loop that can be accommodated by the flexibility of both the poly(A) chain and Fip1 (*A*). However, when a sufficient number of adenosines (probably ∼60) have been added to facilitate Nab2 binding, the reduction in flexibility of the poly(A) tail produced by Nab2 dimerization introduces a stress that detaches Pap1 from Cft1 and Fip1 (*B*). Once detached from Fip1, the processive activity of Pap1 is impaired and it dissociates from the tail, thereby terminating polyadenylation. The termination of polyadenylation then appears to be signaled to the TREX complex via CF1A component Pcf11 to initiate the formation of an export-competent mRNP. The highly schematic representation of the CF1A and polymerase modules of CPF is based on the recent cryo-EM structure of this complex (65).

Once the 3′-UTR RNA is cleaved, polyadenylation starts, catalyzed by poly(A) polymerase that binds the growing end of the poly(A) tail. Poly(A) polymerase is composed of three domains that encircle the active site (69), and *in vivo*, the poly(A) chain is prevented from leaving the enzyme by processivity factors, such as PABPN1 in mammals (70) or CF1 and CPF in yeast (69, 71), that prevent dissociation of the poly(A) tail from the enzyme following the addition of each new nucleotide. In *S. cerevisiae*, Fip1 regulates the activity of poly(A) polymerase (Pap1) through multiple interactions (72) and is thought to be flexible, which would help accommodate the growing poly(A) loop (60, 73, 74) that results from the poly(A) tail being held at both ends (Fig. 5). The processive action of Pap1 requires attachment to the CPF via Fip1 (75) and possibly other components. Polyadenylation is terminated by release of Pap1 from the CPF (67), after which the enzyme ceases to be processive and dissociates from the poly(A) tail. The length of the poly(A) tail is regulated by PABPN1 (for a review, see Ref. 76) and ZC3H14 (77) in higher eukaryotes and Nab2 in *S. cerevisiae* (60). In higher eukaryotes, each PABPN1 chain is thought to bind ∼12–15 adenines (78, 79), and when a sufficient number of PABPN1 molecules are bound, steric factors are proposed to result in the dissociation of poly(A) polymerase from CPSF (66, 67). *In vitro*, PABPN1 forms ∼21-nm-diameter roughly spherical particles when bound to poly(A) RNA that seem to contain about 200–300 nt and so would be attractive candidates for the oligomerization that results in dissociation of poly(A) polymerase from CPSF (78).

Termination of polyadenylation in *S. cerevisiae* is mediated by Nab2 (Fig. 4*A*), which contains seven Zn fingers (76), and could result from either inhibiting Pap1 (by detaching it from Flp1 or from Cft1 or by rephosphorylation of Pta1) or removing the poly(A) tail from Pap1. Nab2 Zn fingers 5–7, which are necessary and sufficient for high-affinity poly(A) RNA binding (80, 81), adopt a conformation in which finger 6 is oriented on the opposite side of the chain to fingers 5 and 7 (54, 80), impairing all three fingers binding simultaneously to a short stretch of a poly(A) chain (Fig. 4*B*). *In vitro*, binding poly(A) RNA induces Nab2 Zn fingers 5–7 to dimerize (Fig. 4*C*), and this feature could provide a mechanism (Fig. 5) analogous to that proposed for PABPN (68, 82) in metazoans for terminating polyadenylation (54). This would propose that, when the poly(A) tail had become sufficiently long to wrap around two Nab2 chains so that they form a dimer, the structure generated would constrain conformation of the poly(A) loop sufficiently to weaken the attachment of Pap1 to Fip1 and CPF (Fig. 5) so that it detaches, after which it ceases to be processive and dissociates from the poly(A) tail. Consistent with this hypothesis, cryo-EM studies (65) of *S. cerevisiae* CPF observed two different objects, either with or without Pap1, indicating that Pap1 can be dissociated from CPF. Once Pap1 has been dissociated from the CPF, it is likely that the transcript is able to dissociate from the complex, freeing the mRNP for nuclear export. In *S. cerevisiae*, it is likely that, following the termination of polyadenylation and dissociation of the CPF from the transcript, conformational changes in the CPF are transmitted to the TREX complex, possibly via CFP component Pcf11. This, in turn, may mediate the attachment and SUMOylation of Yra1 (44, 83) and activation of Sub2 and/or Dbp2, resulting in the attachment of Mex67:Mtr2 and the generation of an export-competent mRNP.

### Questions outstanding

Overall, the mechanism by which transport factors mediate the transport of mature transcripts across the nuclear envelope through nuclear pores and the machinery by which transport factors are released in the cytoplasm are understood in some detail. Major questions outstanding are how transcripts are remodeled to bind transport factors and release adapters and how successful completion of the nuclear processing segment of the gene expression pathway is signaled to initiate generation of export-competent mRNPs.

To enable the export of mRNA through nuclear pores to the cytoplasm, it is necessary both to add nuclear export factors and to release the transcript from RNA polymerase and nuclear processing machinery. After cleavage in the 3′-UTR, the RNA polymerase is still probably attached to the transcript through interactions between its C-terminal domain and the cleavage and polyadenylation machinery that are likely retained until polyadenylation has been completed. Similarly, retention factors appear to impair export until splicing has been completed. Both splicing and polyadenylation appear to be coordinated with the attachment of nuclear export factors, but the temporal sequence of events remains unclear. Indeed, these steps may not necessarily have to occur in a defined sequence, and instead export may only occur when all have been completed. Although most of the factors involved in the generation of export-competent mRNPs have been identified, the precise mechanism by which they function to coordinate the different steps of the nuclear processing machinery is less clear, and a number of aspects remain to be clarified.

(i) Although attachment of nuclear export factors is a prerequisite for movement through nuclear pores to the cytoplasm, it remains unclear how many of these factors are attached to an individual transcript. In higher eukaryotes, the EJC and 5′ cap generate attachment mediated through TREX, whereas completion of polyadenylation, at least in budding yeast, is thought to generate an analogous attachment. It appears that the 5′ and 3′ ends of the transcript are often quite close (85), but it is also possible that a number of export factors are also added along the body of the complex. Although cross-linking and immunoprecipitation studies indicate that ALYREF binds to a region near the 5′ end of the mRNA in a CBP80-dependent manner, they also indicate that it binds near the 3′ end in a PABPN1-dependent manner (86), and other studies (53) indicate binding throughout the coding region. It is not clear how many export factors bind to a given transcript or what features their binding site (or the site to which adapters such as Yra1 bind) might have.

(ii) The termination of polyadenylation appears to be mediated by dissociation of poly(A) polymerase from the cleavage and polyadenylation machinery as a result of steric factors associated with the binding of PABPN1 in higher eukaryotes and Nab2 in budding yeast. *In vitro*, PABPN forms globular aggregates on poly(A) RNA (78) that are proposed to eventually force the dissociation of poly(A) polymerase (for a review, see Ref. 87), but the precise structure of these aggregates needs to be established to define how many PABPN1 chains each contains and how the chains are arranged to generate a defined particle size. In budding yeast, Nab2 clearly controls poly(A) tail length (60) and probably mediates this function through dimer formation (54), but the structure of the Nab2 dimers *in vivo* and whether there are one or two dimers per tail remain to be established.

(iii) Polyadenylation is terminated by the dissociation of poly(A) polymerase from cleavage and polyadenylation machinery, but how this machinery detaches from the 3′-UTR has not been established, although it appears that the generation of export-competent mRNPs by Mex67 binding contributes to this step (88). It may be that the attachment to the 3′-UTR is sufficiently weak to simply dissociate, but it seems more likely that dissociation of poly(A) polymerase generates some conformational change and/or post-transcriptional modification (such as phosphorylation or SUMOylation) in the complex that could also facilitate its release, although detailed evidence for this point is lacking.

(iv) In at least budding yeast, the termination of polyadenylation signals a TREX/Sub2-mediated remodeling of the transcript that results in Yra1 dissociation and Mex67:Mtr2 attachment (Fig. 3). Pcf11 appears to be important in this step (83), but precisely how poly(A) polymerase dissociation influences Pcf11 and how this in turn influences TREX/Sub2 remain obscure.

(v) In budding yeast, there are indications of Mud2 functioning to coordinate splicing and polyadenylation, but the precise mechanism by which this is mediated and the role of other factors that show genetic interactions between these functions are unclear. Similarly, intron-containing transcripts appear to be retained in the nucleus, either at the nuclear basket in budding yeast or in nuclear speckles in higher eukaryotes, and although several components of the machinery involved have been identified, the precise mechanism by which this is mediated remains obscure.

In summary, many of the components of the gene expression pathway that are involved in the nuclear processing and export of transcripts have been identified, but the precise mechanisms by which these steps are coordinated remain to be established. Although the thermal ratchet-based mechanism by which mRNPs are exported to the cytoplasm through nuclear pores has been established in considerable detail, many aspects of the generation of export-competent mRNPs in the nucleus remain unclear. Key checkpoints in the pathway leading to the generation of export-competent mRNPs, which entail establishing that both splicing and polyadenylation have been completed, need to be activated in a timely manner. Providing a comprehensive description of this complex series of integrated processes represents an important challenge and a fruitful area for future studies in this area.

## Footnotes

2 The abbreviations used are:

mRNP: messenger ribonuclear particle
nup: nucleoporin
GAP: GTPase-activating protein
GEF: guanine nucleotide exchange factor
RRM: RNA recognition motif
LRR: leucine-rich repeat
UBA: ubiquitin-associated
RBD: RNA-binding domain
EJC: exon-junction complex
CTE: constitutive transport element
SR protein: RNA-binding protein rich in Ser and Arg
nt: nucleotide(s)
CTF: cleavage and polyadenylation factor
CPSF: cleavage and polyadenylation specificty factor
SUMO: small ubiquitin-like modifier.
